# Assessing the Water Quality of a Stream and Its Relationship with Climate Change Using Water Quality Index and Multivariate Statistical Methods

**DOI:** 10.3390/toxics14060520

**Published:** 2026-06-15

**Authors:** Aslıhan Katip, Elif Demiralp

**Affiliations:** Environmental Engineering Department, Faculty of Engineering, Bursa Uludag University, Bursa 16059, Turkey; elifdemirallp@gmail.com

**Keywords:** climate change, statistical evaluation, water quality index

## Abstract

Industrial and domestic wastewaters, nonpoint pollution sources, and climate change affect stream ecosystems, water quantity, and quality. Within the scope of this study, the water quality of Nilüfer Stream was evaluated using the Water Quality Index (WQI), One-Way ANOVA, the Kruskal–Wallis Test, and Principal Component Analysis (PCA). In the study, 4686 water quality data from seven sampling stations between 2008 and 2024 were used. WQI results showed a distinct decrease in water quality from the upstream to the downstream of the Stream. Average WQI values for the stations were found to be between 140.83 and 487.83. The lowest WQI value was found at Station 1 and the highest WQI value was found at Station 7. According to WQI, the ranking of the stations by magnitude was St7 > St4 > St5 > St6 > St2 > St3 > St1. A statistically significant difference was observed between the stations in terms of WQI, ANOVA, and Kruskal–Wallis Test (*p* < 0.05), and water quality was found to be seasonally diverse. Generally, at stations (except for two stations), the seasonal WQI values ranked by magnitude were autumn > summer > winter > spring. The PCA showed that relationships among parameters originating from industrial wastewater associated with the textile, automotive, and metal industries were stronger (component loadings > 0.75), whereas the groups identified in the upstream basin indicated domestic pollution and agricultural pollution from fertilizers and pesticides. PCA conducted between meteorological parameters and the WQI values of the stations showed that climate change could be effective at only two stations. It was determined that the region located before the wastewater treatment plant (St4) was associated with precipitation, humidity, and evaporation, while the downstream region (St7) was related to wind speed. It was observed that water quality was more influenced by industrial, urban, and agricultural pollution sources than by climate change.

## 1. Introduction

Streams are systems consisting of a main stem and various tributaries, fed by surface runoff, spring waters, seepages, and the melting of snow and glaciers [1,2]. The most critical parameters influencing changes in a stream’s water quality can vary from each stream and may vary along the stream due to diverse environmental conditions and anthropogenic activities [3]. Simultaneously, seasonal variations observed in meteorological and hydrological conditions exert a significant impact on water quality [4]. Discharge of industrial and urban wastewaters and agricultural activities (fertilizers) are important sources of pollution for streams. However, since climate change is a function of many factors such as precipitation, surface runoff of the stream basin, and groundwater flow, the seasonal variations in these factors have a strong influence on flow rates and, consequently, on the concentration of pollutants in the stream [5]. As a result of regular monitoring of the stream due to spatial and temporal variations in water quality, a complex, large data matrix containing numerous parameters that are generally difficult to understand is formed [6]. As surface water quality data may exhibit non-normal distribution, employing parametric and non-parametric methods intended for normal and non-normal distribution in statistical analyses can yield accurate results [7,8]. To provide an understandable and effective assessment in water quality studies, water quality indices and multivariate statistical methods are employed. The Water Quality Index (WQI) is an integrated indicator that allows the quality status of water to be presented in an easily understandable manner by having various water quality parameters aggregated and reduced to a single numerical value [9,10]. The use of WQI simplifies the presentation by summarizing the results of numerous parameters associated with the water resource [11,12]. WQI was first proposed and designed by Horton (1965) [13]. Subsequently, various versions of WQI models have been developed and applied [14,15,16,17]. Depending on geographical conditions and institutional standards, there are many different indices in the literature, such as the National Sanitation Foundation Water Quality Index (NSFWQI), Weighted Arithmetic Water Quality Index (WAWQI), Oregon Water Quality Index (OWQI), and Canadian Council of Ministers of the Environment Water Quality Index (CCMEWQI). WQI is widely used in surface and groundwater during the processes of sustainable management and monitoring of water resources [18,19,20].

Principal Component Analysis (PCA), one of the statistical methods, is widely used in many studies to gain insight into the factors affecting water quality [21,22,23]. This method assists in evaluating the effects of hydraulic and meteorological conditions on water quality and determining the factors influencing changes in water quality parameters [4]. PCA is an effective method for simplifying the water quality dataset, understanding the relationships between indicators, and identifying the most informative features in the dataset [24]. In studies conducted in the Danube, Mudan River Watershed, and Annaba, the spatial and temporal variability of water quality was analyzed by applying the Water Quality Index (WQI) using different parameters and performing Principal Component Analysis (PCA) [25,26,27]. In a study conducted in the Basho Valley, water quality was assessed through PCA, WQI, and GIS methods, and it was identified that anthropogenic factors had a significant impact on water quality [28].

Among the main driving mechanisms of decades-long water quality changes are hydroclimatic variations and increases in water and air temperatures. These effects are further increasing with other anthropogenic factors. It shows that stream water quality is generally deteriorated at a rate of 68% by droughts and heat waves, 51% by heavy rainfall and floods, and 56% by long-term climate change [29]. Understanding threshold values and dynamic changes arising from synergistic effects within hydrological systems remains challenging. In recent years, statistical and artificial intelligence models demonstrating the impact of meteorological factors on water quality have begun to be used [30]. In Japan, statistical models were used to investigate the relationship between climatic factors, precipitation, air temperature, and physicochemical water quality parameters in the Kiso River. In particular, it has enabled the understanding of interactions between water and environmental parameters in regions where data availability is limited [31]. However, the data used in these models are generally short-term [30]. This study is important for statistically determining the relationships between WQI and parameters, for making accurate water quality predictions using a limited number of determining parameters due to laboratory and field study costs, for conducting more informed monitoring in regions with insufficient data, and for making statistical predictions based on climatic information. This study provides information different from that of other studies for the development of tools and techniques supporting the design of robust water quality management strategies based on long-term data in streams receiving intensive industrial and domestic wastewater inputs, in a world confronted with frequent and severe hydroclimatic extremes. The mixing of wastewaters with different characteristics and extreme meteorological conditions increases the toxicity of substances present in water and may make the water more hazardous to human and environmental health by restricting its intended uses [29,32]. Therefore, investigating the relationships between water quality and meteorological parameters within the scope of this study will provide a scientifically significant contribution.

The Nilüfer Stream is a heavily polluted stream by agricultural activities, domestic and industrial wastewater [33,34,35]. Furthermore, it has been determined that atmospheric changes resulting from global climate change affect the water quality dynamics of the Doğancı Dam on the Nilüfer Stream [36]. Within the scope of this study, by using One-way ANOVA (Analysis of Variance), Kruskal–Wallis test, Spearman Correlation Analysis and Principal Component Analysis methods, the interrelationships among parameters affecting water quality, the uncovering of the similarities and differences between stations, and potential pollution sources were tried to be understood. Furthermore, the water quality status, which was determined according to concentration levels, national and international standard values, was evaluated more comprehensively by using many physicochemical parameters together, via the WQI method. In addition, pollution levels were statistically correlated with meteorological parameters to assess the impact of climatic factors on stream quality. The aim of this study is to determine water quality in heavily polluted streams using a limited number of key parameters measured over long-term periods, and to evaluate the effects of climatic factors on water quality using statistical methods.

## 2. Materials and Methods

### 2.1. Study Area and Data

The Nilüfer Stream, located within the boundaries of Bursa, originates from the slopes of Uludağ. As a sub-basin of the Susurluk Basin, the Nilüfer Stream is 168 km long and there are many tributaries [37,38]. The average flow discharge of the Nilüfer Stream is 23.58 m^3^/s [39,40]. However, this value varies between 0.178 m^3^/s and 23.58 m^3^/s at different tributaries and flow measurement points [40]. Since the Nilüfer Stream does not possess a natural flow regime, a controlled discharge is released from the Doğancı Dam. In addition, discharge measurements are not conducted at every location along the stream. The stream flows northwestward through a valley and empties into the Marmara Sea through the Karacabey Strait [39].

There are two municipal wastewater treatment plants along the Nilüfer Stream: the West Wastewater Treatment Plant (WWWTP) (87,500 m^3^/day) and the East Wastewater Treatment Plant (EWWTP) (240,000 m^3^/day) [41]. Along the river, there are many organized industrial zones (OIZs), namely Nilüfer OIZ (792 m^3^/day), Bursa OIZ (96,000 m^3^/day), Demirtaş OIZ (82,500 m^3^/day), Kestel OIZ (100,000 m^3^/day), Uludağ OIZ (100,000 m^3^/day), Hasanağa OIZ (7000 m^3^/day), and Kayapa OIZ [42]. Wastewaters originating from the Uludağ (Gürsu) OIZ, Barakfakih OIZ and Kestel OIZ are treated by the S.S. Yeşil Çevre Services and Management Cooperative (100,000 m^3^/day) and discharged into the stream [42,43]. The Nilüfer Stream is used as an irrigation water source for agricultural lands in Bursa Province and also serves as a receiving environment for wastewater discharges. As one of the country’s production centers, Bursa Province is important for agriculture and industry and is mostly composed of the metal, automotive, textile, and food sectors [44,45].

**Figure 1:**
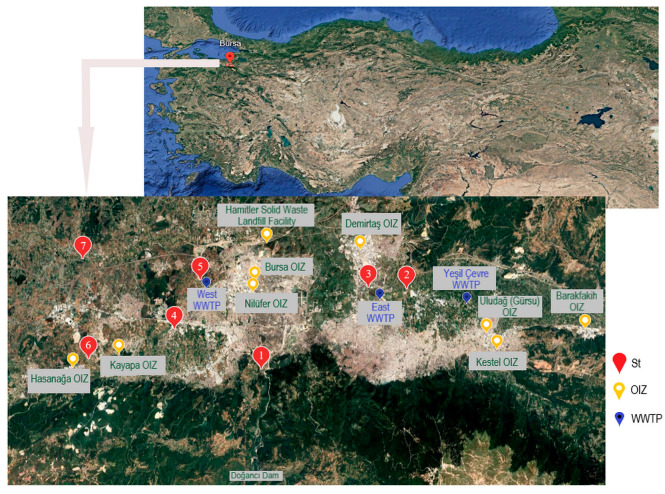
Satellite view of Nilüfer Stream and sampling stations (acquired from Google Earth on 24 March 2026) [46].

Along the Nilüfer Stream, a total of seven sampling stations were identified from upstream to downstream, including two on the main channel and five on the tributaries (Figure 1). The first sampling station on the Nilüfer Stream (Station 1) was considered the upstream point, whereas the last sampling station (Station 7) was considered the downstream outlet point. The sampling stations and features were presented in Table 1.

In this study, water quality parameter data obtained from water samples collected by the General Directorate of Bursa Water and Sewerage Administration (BWSA) from seven sampling stations located on the Nilüfer Stream were used [47]. The water quality data consist of seasonal mean values measured over the period 2008–2024. The parameters measured before 2008 do not exhibit temporal continuity, and measurements are not available for each station considered in this study. In order to ensure accurate statistical analyses and to construct long-term water quality data, the dataset covering the period 2008–2024 was selected for this study [48]. For meteorological parameters, data obtained from the Bursa Meteorology Directorate for the Central Bursa Station covering the period from 2008 to 2024 were used [49]. For the calculation of the Nilüfer Stream water quality index, concentrations of pH, electrical conductivity (µS/cm), dissolved oxygen (mg/L), COD (mg/L), TN (mg/L), TP (mg/L), Pb (mg/L), Ni (mg/L), Cu (mg/L), Cd (mg/L), and Zn (mg/L) were used Şimşek et al., (2022) [50]. Water quality parameters were analyzed in the BWSA laboratory, which is accredited by TS EN ISO/IEC 17025:2017 [51]. In this study, a 17-year-long-term dataset was used. To ensure parameter continuity and conduct cost-effective laboratory analysis, total nitrogen (TN) and total phosphorus (TP) were selected as nutrient parameters using a tiered testing approach [52,53]. In the Nilüfer Stream, which has been monitored over many years, inorganic nitrogen could not be measured at every sampling location. In addition, these parameters are also used in the Water Quality Index (WQI) formula applied within the scope of this study. A staged analytical approach was applied to determine total nitrogen (TN) as a single parameter in surface waters and wastewater, combining chromatographic analysis of inorganic anions (930 Compact IC Flex, Metrohm AG, Herisau, Switzerland) (TS EN ISO 10304-1) [54] with macro-Kjeldahl digestion (Kjeltec 8400, FOSS Analytical A/S, Hillerød, Denmark) (SM 4500-Norg B) [55]. The pH values of the samples were determined using a pH meter via the electrometric method (sensION+ MM156, Hach Company, Loveland, CO, USA) (SM 4500-H^+^ B) [55]. The electrical conductivity (EC) of the samples was measured using a conductivity meter (sensION+ MM156, Hach Company, Loveland, CO, USA) according to the Conductivity—Laboratory Method (SM 2510 B) [55]. Chemical Oxygen Demand (COD) was determined by the open reflux titrimetric method using potassium dichromate oxidation (Merck KGaA, Darmstadt, Germany) (SM 5220 B) [55]. Total phosphorus (TP) concentrations were determined by spectrophotometry following persulfate digestion and the ascorbic acid method (DR3900, Hach Company, Loveland, CO, USA) (SM 4500-P E) [55]. Dissolved oxygen (DO) levels of the samples were measured using the membrane electrode method (sensION+ MM156, Hach Company, Loveland, CO, USA) (SM 4500-O G) [55]. The heavy metal analysis (Cd, Cu, Ni, Pb, Zn) was performed using the ICP-OES technique (Varian Inc., Palo Alto, CA, USA) (EPA Method 6010D) after a microwave-assisted closed-vessel acid digestion pretreatment (MARS 5, CEM Corporation, Matthews, NC, USA) (EPA Method 3015A) [56,57]. To ensure data reliability and accuracy, quality assurance and quality control procedures, including analysis of procedural blanks, calibration using certified multi-element standard solutions, periodic instrument performance verification, and replicate analyses, were applied. The relative standard deviation (RSD) for replicate measurements was kept below 5%. To ensure the accuracy of the calibration and standardization procedure, blank samples and European standard reference materials (ERM-CA713) (LGC Standards GmbH, Wesel, Germany) were used in each digestion batch, and all analyses were performed in duplicate [58]. ICP Multi-Element Standard Solution IV (Certipur, Merck KGaA, Darmstadt, Germany) was used for device calibration and temperature control was applied by maintaining the laboratory at 25 °C to minimize potential thermal expansion during the calibration process [59,60]. The water quality parameters used in the water quality index, which considers priority parameters for global and local aquatic ecosystems and human health [50,61], were selected by examining the parameters included in the water quality standards of Turkey, WHO, and EPA. In collecting seasonal data, three replicate water samples were collected from each sampling station in three different months for each season [62,63].

A total of 4686 data points were used in this study, and missing values were completed using the Multiple Imputation (MI) method with IBM SPSS 22.0 software [64]. Multiple imputation is a method of completing datasets by generating multiple sets of plausible values in order to preserve variability within the data [65]. Among the generated datasets, the completed dataset that best captures the overall data structure was selected. Subsequently, outliers that could cause biases in the overall dataset were removed from the dataset [66,67].

Meteorological data consistency was checked through spatial consistency analysis [68]. In addition, internal theoretical consistency was evaluated, and the data were verified against physical laws [68,69]. Meteorological data were recorded daily. It was also assessed whether the observed values fell within the historical climatic limits of the study region (gross error—range check method) [69]. The meteorological dataset contained a small amount of missing data. As with the water quality data, missing values were completed using the Multiple Imputation method. Monthly averages of the meteorological parameters pressure (hPa), relative humidity (%), air temperature (°C), wind speed (m/s), precipitation (mm), evaporation (mm), and snow depth (cm) were selected for the assessment of the effects of climate change [70,71].

### 2.2. Water Quality Index (WQI) Calculations

Due to climate change, summers in Bursa are longer and warmer, and the warm period may last from early June to mid-September [72]. Therefore, the period from 1 June to 15 September was defined as summer, 15 September to 15 December as autumn, 15 December to 15 March as winter, and 15 March to 1 June as spring. The prolongation of non-rainy periods may lead to seasonal drought conditions. Snowy days in winter are also decreasing. Snowy days in winter have also decreased. This situation leads to changes in temporal planning for water management. Therefore, the Water Quality Index (WQI) calculations were performed using seasonal and monthly mean values.

The number of parameters used to evaluate water quality in Water Quality Indices (WQI) varies considerably across models, and there is no specific rule or guideline for which parameters should be included [73]. In this study, for the water quality index used, 11 parameters (pH, conductivity, DO, COD, TN, TP, Ni, Zn, Cu, Pb and Cd) were selected from physicochemical parameters and heavy metal parameters representing water quality, and the water quality index was applied. This index was applied in a way that includes the parameters of the Nilüfer Stream that reflect local pollution sources such as organic matter and metals. A weight (w_i_) was assigned to each parameter by examining the calculations presented in previous studies. Weights ranging from 1 to 5 (from lowest to highest) were assigned to the parameters to express them on a simple, standardized scale, taking into account their relative impacts on water quality and human health [50,74]. A value of 5 was assigned to EC, TN, and TP parameters due to their importance as key indicators of domestic, industrial, and agricultural pollution in water quality assessment. A value of 4 was assigned to pH, as it affects many chemical and biological processes and water usability; to COD, as it is a significant indicator of organic pollution and is widely used in water quality assessment; and to Pb, due to its high toxicity and high risk to human and environmental health. A weight value of 3 was assigned to DO because of its critical role in maintaining the health of aquatic ecosystems and its recognition as an indicator of overall water quality status, and to Ni because, although toxic, it is generally considered to pose a lower risk than Pb. Zn was assigned a weight value of 1 as it is generally regarded as one of the least toxic heavy metals. Cu was assigned a weight value of 2 because it is an essential trace element for living organisms, but may become harmful at elevated concentrations, while Cd was also assigned a weight value of 2 because, despite its high toxicity, it generally occurs at lower levels than other heavy metals [50,61]. The WQI was calculated using the mathematical formulas listed below. This index model is widely used to evaluate water quality in various countries [75,76,77].

(1) $$W_{i}=\frac{w_{i}}{\sum_{i=1}^{n}w_{i}}$$

In Equation (1), the number of parameters is denoted by n, while w_i_ represents the weight coefficient assigned to the parameters, and W_i_ represents the calculated relative weight.

(2) Qi=CiSi×100

In Equation (2), Q_i_ represents the quality rating, C_i_ represents the measured concentrations of each parameter. S_i_ represents the standard values of the chemical parameters recommended in the Turkish Surface Water Quality Regulation (TSWQR) and n represents the number of parameters [50]. The water quality index (WQI), which is equal to the sum of the products of the calculated relative weight (W_i_) and the quality rating (Q_i_), is expressed in Equation (3).

(3) $$\mathrm{WQI}=\sum W_{i}\times Q_{i}$$

The water quality index values were classified into five categories: WQI < 50 as excellent (suitable for drinking, irrigation, and industrial use), between 50 and 100 as good (suitable for drinking, irrigation, and industrial use), between 100 and 200 as poor (suitable for irrigation and industrial use), between 200 and 300 as very poor (suitable only for irrigation), and WQI > 300 as unsuitable [16,78,79,80,81]. A lower WQI value indicates better water quality. This classification represents the overall condition of the water body and its suitability for various uses such as drinking, agriculture, and industry [82]. There is no single standard ranking system for water quality classification based on WQI scores [83].

### 2.3. One-Way ANOVA and Kruskal–Wallis Test

Analysis of variance (ANOVA) is an analytical method that tests the statistical significance of differences in group means by partitioning the total variability in a dataset into systematic and random components [84]. The null hypothesis, which assumes that all means are equal, is rejected when the *p*-value is small (*p* < 0.05), as the probability of the hypothesis being true decreases [85]. While the ANOVA test depends on verifying the assumptions of normality and homogeneity of variances, the Kruskal–Wallis test allows comparisons between groups independently of these assumptions [86]. The Kruskal–Wallis test is preferred as an alternative to the One-Way ANOVA test [87,88]. The significance of differences among the seven stations was tested using One-way analysis of variance (ANOVA) along with the Tukey post hoc test [89,90]. The analyses were performed using IBM SPSS Statistics 22.0 (2013) [64]. Prior to the ANOVA, the assumption of normality was assessed using the Kolmogorov–Smirnov test [91], which indicated that the raw data were not normally distributed (*p* < 0.05). Accordingly, to improve data symmetry, each measurement value was transformed using the log_10_(x) function, and the normality assumption was subsequently satisfied (*p* > 0.05) for all stations except Station 1 [92]. For this station where normality could not be achieved, examination of the histogram showed a rightward skewed (positive skewness) distribution. The box plot was also evaluated and no extreme outliers were detected. Studies show that ANOVA can be applied even if the data deviate slightly [93]. In this case, One-way ANOVA was applied due to the fact that the sample size is large, as well as the fulfillment of the homogeneity of variances assumption (Levene’s test) [94]. In addition, considering the complex nature of water quality data, the non-compliance of the normality assumption in one station may raise concerns about the reliability of the results; therefore, the Kruskal–Wallis test was also applied [85,95]. The Kruskal–Wallis test is a non-parametric, rank-based statistical method used to determine whether there are significant differences among independent groups [96]. A *p*-value of less than 0.05 (*p* < 0.05) is considered indicative of a statistically significant difference among the groups [97]. When significant differences are detected, multiple comparison (post hoc) analyses are performed to identify the specific groups that differ from one another.

### 2.4. Spearman Correlation Analysis

The Spearman correlation analysis is a non-parametric statistical technique used to assess the strength and direction of the association between two variables [98]. It is known that water quality data generally do not meet the assumption of normality [99]. Since the data did not meet the normality assumption, Spearman correlation analysis was performed instead of parametric correlation methods. The significance levels were assessed based on *p* < 0.05 [100].

### 2.5. Principal Component Analysis (PCA)

Principal component analysis (PCA) is a method that aims to summarize the information in an original dataset by generating new, uncorrelated variables (principal components) from a high-dimensional dataset [24,101]. In the PCA method, eigenvalues are used to identify the principal components (PCs), and components with eigenvalues greater than 1 are considered for evaluation [101,102]. The principal component (PC) is defined as a linear combination of the original variables and can be written as follows:

(4) $$z_{\mathrm{ij}\;}=a_{i1}x_{1j}+a_{i2}x_{2j}+a_{i3}x_{3j}+\cdots +a_{\mathrm{im}}x_{\mathrm{mj}}$$

In this equation, z denotes the component score, a represents the component loading, and x is the measured value of the variable. The indices i refer to the component number, j refers to the sample number, and m indicates the total number of variables [1]. In Principal Component Analysis (PCA), the first principal component represents the largest variance in the dataset, and the higher the eigenvalue of a component, the more important it is for the dataset [103]. The projections of the original variables onto the principal component space (loadings) are related to the correlations between the principal components and the variables [5]. The suitability of the dataset for PCA is assessed using the Kaiser–Meyer–Olkin (KMO) measure and Bartlett’s test of sphericity. A KMO value greater than 0.5 and a statistically significant Bartlett’s test result (*p* < 0.05) indicate that the dataset is suitable for analysis [6,74]. In PCA, varimax rotation is widely used to facilitate the interpretation of results [104,105,106]. In interpreting component loadings, the absolute value of the coefficient indicates the strength of the relationship between the variable and the component, whereas the sign indicates the direction of the linear relationship (positive or negative). These loadings indicate the contribution of each parameter to a given component and are used to interpret underlying pollution sources or water quality factors [103]. In PCA, component loadings were classified as strong (│loading│ > 0.75), moderate (0.50–0.75), and weak (0.30–0.50) associations [107].

In this study, PCA was applied using various approaches: meteorological and water quality parameters were analyzed separately, and meteorological parameters were analyzed together with water quality index data. Statistical analyses were performed using IBM SPSS Statistics 22.0 (2013) [64]. Since the parameters must have a common unit of measurement, all data were standardized (using z-score values) before analysis [6]. Kaiser–Meyer–Olkin (KMO) and Bartlett’s tests of sphericity were conducted to assess the suitability of the data for analysis. The neglect of small loadings in a PC and the expression of the component as a combination of the remaining variables are widely used and are subjective [108]. In the PCA, the parameters contributing most to the principal component were selected as those with loading values greater than 0.30 (positive or negative).

## 3. Results

**Figure 2:**
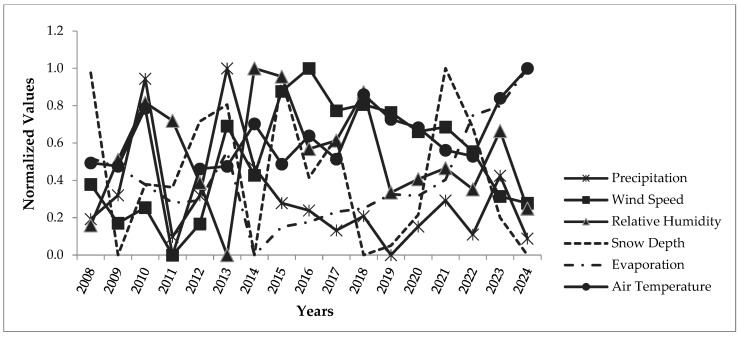
Graph of interannual variations in the normalized meteorological parameters.

The mean and standard deviation values of the meteorological parameters of Bursa Province for the 2008–2024 period were listed in Table 2. When Table 2 was examined, the average monthly air temperature was 15.72 °C and the average monthly precipitation was 1.9 mm. When the interannual variations in meteorological data are examined, an increasing trend in air temperature and evaporation over the years is observed, whereas precipitation and snow depth exhibited irregular interannual variability. Wind speed, relative humidity, and atmospheric pressure showed generally smaller variations. The graph of interannual variations in the normalized meteorological parameters was shown in Figure 2.

The mean and standard deviation values of the water quality parameters for the period 2008–2024 were listed in Table 3. When Table 3 is examined, the pollution rankings of the stations based on conventional parameters are as follows: EC for 6 > 2 > 4 > 3 > 5 > 7 > 1; DO for 1 > 5 > 4 > 2 > 3 > 6 > 7; TN for 4 > 7 > 5 > 6 > 2 > 3 > 1; TP for 7 > 3 > 6 > 2 > 5 > 4 > 1; and COD for 2 > 6 > 3 > 7 > 4 > 5 > 1. Overall, Station 1 was observed to have the lowest concentrations. It was determined that Stations 7 for TP, 4 for TN, 6 for EC, and 2 for COD exhibited the highest pollution levels. When the pollution rankings of the stations based on heavy metals are examined, the order is as follows: Pb for 5 > 4 > 1 > 7 > 2 > 6 > 3; Ni for 4 > 5 > 7 > 6 > 2 > 3 > 1; Zn for 6 > 4 > 5 > 7 > 2 > 3 > 1; Cu for 7 > 4 > 2 > 5 > 1 > 3 > 6; and Cd for 5 > 2 > 1 > 4 > 7 > 3 > 6. It was observed that, at many stations, the standard deviation of heavy metal concentrations exceeded the mean.

### 3.1. Results of Water Quality Index (WQI) Calculation 

When the overall mean WQI values of the Nilüfer Stream for the period 2008–2024 are examined, the water quality at Station 1, with a value of 140.83, was classified as poor quality (100–200), that is, suitable for irrigation and industrial use, whereas the other stations were classified as unsuitable (>300), meaning that they are not suitable for use. The ranking of stations according to WQI values is as follows: Station 7 > Station 4 > Station 5 > Station 6 > Station 2 > Station 3 > Station 1. The seasonal and overall water quality index (WQI) values for each station were presented in Table 4.

### 3.2. One-Way ANOVA Results

To test the differences among the seven stations, a parametric ANOVA was conducted, and Levene’s test result was 0.370. In this case, since the *p*-value was greater than 0.05, the assumption of homogeneity of variances was found to be satisfied. Therefore, a classical one-way ANOVA was applied. The results of the one-way ANOVA were presented in Table 5. The analysis revealed a statistically significant difference in the water quality index among the stations (*p* < 0.05).

The results of the Tukey post hoc test, conducted to identify which stations differ, were examined, and station pairs with *p* < 0.05 were considered to show statistically significant differences. The results have shown that Station 1 is significantly different from all other stations, Station 2 differs significantly only from Station 7, and Station 7, in addition to Stations 1 and 2, also differs significantly from Station 5, while the other stations (3, 4, and 6) do not show statistically significant differences.

### 3.3. Kruskal–Wallis Test Results

**Figure 3:**
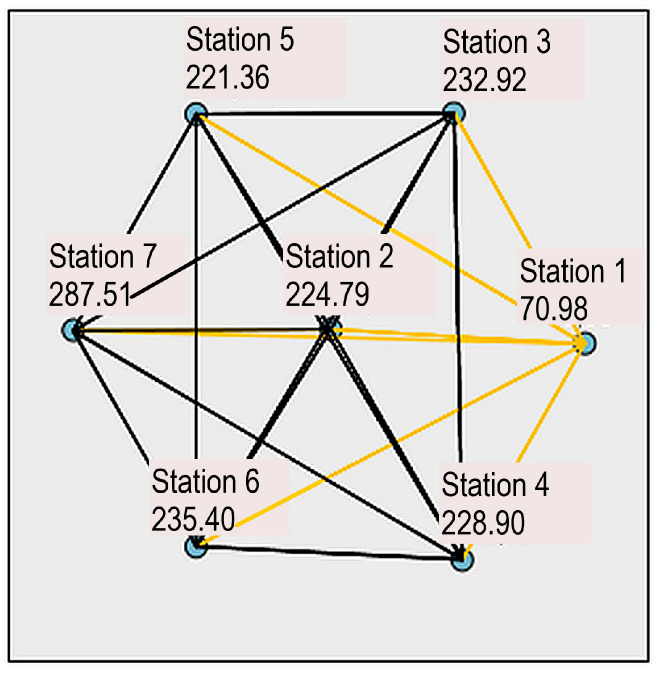
Node diagram of pairwise comparisons.

The test results indicated a statistically significant difference among the stations (*p* < 0.05). The mean ranks for each station showed that Station 1 had the lowest, whereas Station 7 had the highest. The pairwise comparisons conducted to identify significantly different stations showed that Station 1 was significantly different from all other stations. On the other hand, no statistically significant differences were observed among the remaining stations. The node diagram of pairwise comparisons is presented in Figure 3. When the findings obtained from the ANOVA and Kruskal–Wallis tests are examined, it has been demonstrated that there are statistically significant differences among the stations (*p* < 0.05).

### 3.4. Spearman Correlation and Principal Component Analyses

For the analysis conducted using meteorological parameters, results of KMO = 0.796 and Bartlett’s test of sphericity = 0.000 (*p* < 0.05) were obtained. These results indicate that the dataset is suitable for analysis and that significant relationships exist among the variables. The results of the principal component analyses applied to meteorological parameters, water quality parameters, and the combined meteorological parameters and water quality index (WQI) values and spearman correlation analysis were presented in Table 6. As a result of the principal component analysis, it was observed that two principal components with eigenvalues greater than 1 explained 68.86% of the total variance (Table 6). PC1 alone accounted for 50.59% of the variance, indicating that a substantial portion of the data variability is captured by this component. PC2 accounted for 18.26% of the total variance. PC1 exhibited strong negative loadings (inverse relationship) for air temperature (−0.895) and evaporation (−0.884) with component loadings > 0.75, strong positive loadings (direct relationship) for relative humidity (0.894) and atmospheric pressure (0.795), and weak positive loadings for precipitation (0.472) within the range of 0.30–0.50. PC2 showed a strong positive loading for snow depth (0.770) and a moderate positive loading for wind speed (0.722) within the range of 0.50–0.75.

For the analysis using meteorological parameters together with the water quality index values of the stations, the KMO value was found to be 0.595 and Bartlett’s test was significant (*p* < 0.05). For the analysis using water quality parameters, the KMO values for the stations ranged between 0.550 and 0.771, and Bartlett’s test of sphericity was significant (*p* < 0.05). The results for each station indicated that the datasets were suitable for analysis and that significant relationships existed among the variables.

PCA applied to meteorological data and station WQI values revealed that five components with eigenvalues greater than 1 explained 77.78% of the total variance. PC1 accounted for 21.34% of the variance and showed strong positive loadings (component loadings > 0.75) for WQI-St 1, WQI-St 2, and WQI-St 6. PC2 explained 20.82% of the total variance and showed strong positive loadings for actual pressure and snow depth, and a moderate negative loading (0.50–0.75) for air temperature. PC3 accounted for 17.65% of the total variance and exhibited strong positive loadings for precipitation and relative humidity, a moderate negative loading for evaporation, and a moderate positive loading for WQI-St 4. PC4 explained 9.73% of the total variance and showed a strong negative loading for WQI-St 7 and a moderate positive loading for wind speed. PC5 accounted for 8.24% of the total variance and showed a strong positive loading for WQI-St 3 and a moderate positive loading for WQI-St 5. The scree plot of principal component analysis for meteorological parameters and stations’ WQI values was given in Figure S1.

**Figure 4:**
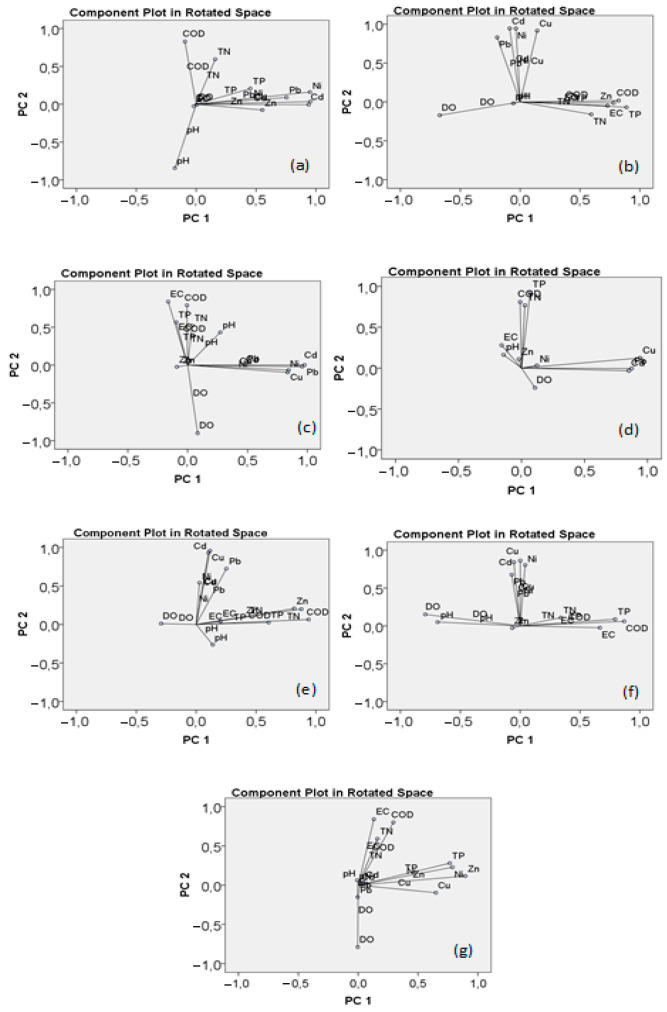
PCA biplots of principal component analysis for water quality parameters at the stations (Station 1 (**a**), Station 2 (**b**), Station 3 (**c**), Station 4 (**d**), Station 5 (**e**), Station 6 (**f**), Station 7 (**g**)).

The results of PCA applied to water quality parameters indicated that four components with eigenvalues greater than 1 for Station 1 explained 78.30% of the total variance (Table 6). PC1 alone accounted for 34.95% of the variance and showed strong positive loadings (component loadings > 0.75) for Cu, Ni, and Cd, and moderate positive loadings (0.50–0.75) for Pb and Zn. PC2 explained 16.72% of the total variance and showed a strong negative loading for pH and a strong positive loading for COD. PC3 accounted for 15.80% of the total variance and exhibited a strong negative loading for DO and moderate positive loadings for TN and TP. PC4 explained 10.82% of the total variance and showed a strong positive loading for EC. For Station 2, PCA results indicated that three components with eigenvalues greater than 1 explained 73.61% of the total variance. PC1 alone accounted for 31.66% of the variance and showed strong positive loadings for TP, COD, and Zn, moderate positive loadings for EC and TN, and a moderate negative loading for DO. PC2 explained 30.69% of the total variance and showed strong positive loadings for Cd, Ni, Cu, and Pb. PC3 accounted for 11.25% of the total variance and showed a strong positive loading for pH. For Station 3, PCA results indicated that three components with eigenvalues greater than 1 explained 73.62% of the total variance. PC1 accounted for 30.93% of the variance and showed strong positive loadings for Pb, Cd, Cu, and Ni. PC2 explained 26.66% of the total variance and exhibited a strong negative loading for DO, strong positive loadings for EC and COD, and a weak positive loading (0.30–0.50) for pH. PC3 accounted for 16.02% of the total variance and showed a strong positive loading for Zn and moderate positive loadings for TP and TN. For Station 4, PCA results indicated that four components with eigenvalues greater than 1 explained 74.27% of the total variance. PC1 accounted for 22.28% of the variance and showed strong positive loadings for Cu, Pb, and Cd. PC2 explained 20.86% of the total variance and showed strong positive loadings for TP, COD, and TN. PC3 accounted for 18.76% of the total variance and exhibited a strong negative loading for DO and moderate positive loadings for EC and Zn. PC4 explained 12.36% of the total variance and showed a strong positive loading for pH and a moderate positive loading for Ni. For Station 5, PCA results indicated that three components with eigenvalues greater than 1 explained 68.53% of the total variance. PC1 accounted for 26.61% of the variance and showed strong positive loadings for COD, TN, and Zn, and a moderate positive loading for TP. PC2 explained 25.14% of the total variance and showed strong positive loadings for Cu and Cd and a moderate positive loading for Pb. PC3 accounted for 16.77% of the total variance and exhibited a strong positive loading for EC, a moderate negative loading for DO, a moderate positive loading for Ni, and a weak positive loading for pH. For Station 6, PCA results indicated that three components with eigenvalues greater than 1 explained 65.13% of the total variance. PC1 accounted for 27.84% of the variance. It showed strong positive loadings for COD and TP, a moderate positive loading for EC, a strong negative loading for DO, and a moderate negative loading for pH. PC2 explained 23.73% of the total variance and showed strong positive loadings for Cu, Cd, and Ni, and a moderate positive loading for Pb. PC3 accounted for 13.55% of the total variance and showed a strong positive loading for Zn and a moderate positive loading for TN. The scree plots of principal component analysis for water quality parameters at the stations (Station 1 (a), Station 2 (b), Station 3 (c), Station 4 (d), Station 5 (e), Station 6 (f), Station 7 (g)) were given in Figure S2. For Station 7, PCA results indicated that four components with eigenvalues greater than 1 explained 78.78% of the total variance. PC1 accounted for 23.29% of the variance and showed strong positive loadings for Zn, Ni, and TP. PC2 explained 22.75% of the total variance and showed strong positive loadings for EC and COD, a strong negative loading for DO, and a moderate positive loading for TN. PC3 accounted for 21.07% of the total variance and showed strong positive loadings for Cd and Pb and a moderate positive loading for Cu. PC4 explained 11.66% of the total variance and showed a strong positive loading for pH. The PCA biplots of principal component analysis for water quality parameters at the stations (Station 1 (a), Station 2 (b), Station 3 (c), Station 4 (d), Station 5 (e), Station 6 (f), Station 7 (g)) were presented in Figure 4. 

The relationships found in PCA across all stations were found to be consistent with the correlation coefficients determined in Spearman correlation analyses. 

## 4. Discussion

According to Table 3, the parameter values at the stations exceeded the drinking and aquatic life water limits established by the WHO and USEPA [109,110]. In addition, based on the TSWQR classification, Station 1 was assigned to Class II, while the other stations were assigned to Class III. Class II waters (with potential for use as drinking water) can be used for recreational purposes, for fish production excluding trout, and as irrigation water provided that irrigation water quality criteria are met, whereas Class III waters (polluted waters) can be used for aquaculture and industrial purposes after appropriate treatment, except for facilities requiring high-quality water such as food and textile industries [111].

The high mean EC and COD values at Stations 6 and 2 indicate elevated levels of chemical pollution at these locations. Discharges from organized industrial zones (Hasanağa, Kayapa, Uludağ, and Kestel) and nearby food processing plants surrounding Stations 6 and 2 contribute to the increased concentrations of these parameters in the stream. At Station 7, the high TP and TN values together with low DO levels indicate that there may be a substantial load of biodegradable organic matter in this area. In addition, the increase in TN and TP is attributed to agricultural activities in the region [44]. The high standard deviations observed at these stations may reflect temporally variable pollution levels influenced by irregular discharge patterns. The distribution of heavy metals in the Nilüfer Stream reaches high levels, particularly at Stations 4 and 5 (Pb, Ni, Zn, Cd) and Station 6 (Zn), which correspond to discharge points of tributaries influenced by industrial activities. This situation is consistent with the pollution profile associated with industrial activities such as metal processing and automotive industries in organized industrial zones (NOSAB, Hasanağa OSB, and Kayapa OSB), which are known sources of heavy metal contamination (Pb, Ni, Zn) [112]. Elevated concentrations of Cu and Ni at Station 7 suggest the cumulative impact of pollution inputs from multiple industrial sources. Furthermore, the fact that the standard deviation values of heavy metal concentrations exceed the mean values at many stations indicates that pollutant loads are not homogeneous and are largely variable and point source dominated inputs.

### 4.1. Evaluation of Water Quality Index (WQI)

**Figure 5:**
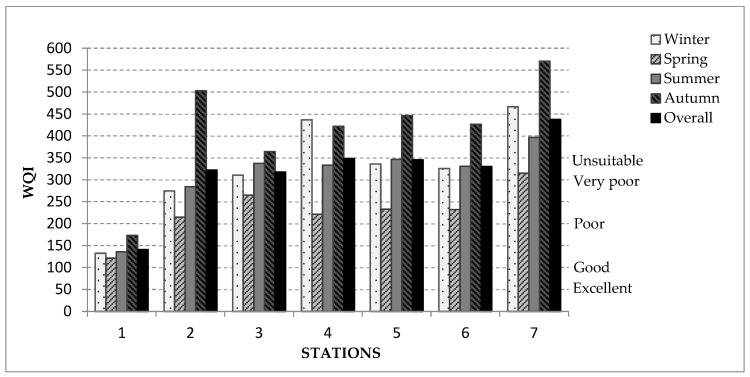
Seasonal water quality index values by station.

The Water Quality Index (WQI) results showed that most stations fell into the poor quality (suitable for irrigation and industrial use) and unsuitable water quality classes according to the applied WQI classification. Among the stations, Station 1, located upstream and relatively unaffected by direct industrial impacts, exhibited the best water quality, with the lowest WQI (140.83), whereas Station 7 (downstream) showed the worst water quality, with the highest WQI (437.83). These results indicate a clear deterioration in water quality from upstream to downstream. Station 1 was classified as suitable for irrigation, while all other stations were deemed unsuitable for irrigation. The ranking of WQI values among stations was 7 > 4 > 5 > 6 > 2 > 3 > 1, with Station 7 having the poorest water quality. The unsuitable water quality class at Station 7 is attributed to its downstream location, where pollutant loads from all upstream stations accumulate [113]. It is observed that the tributaries where Stations 4 and 5 are located are more polluted compared to the other tributary stations. This is attributed to point sources from surrounding urban and industrial areas (Nilüfer OSB, Bursa OSB, and small-scale industries) and to agricultural activities. Domestic and industrial wastewater originating from small and medium scale industrial facilities in the Çalı, Yaylacık, and Ürünlü areas reaches Station 4. At Station 5, in addition to the effluent from the Bursa West Wastewater Treatment Plant, where all domestic wastewater from the western part of the city is treated, domestic and industrial wastewater from Nilüfer OIZ and Bursa OIZ also reaches the station. Seasonal water quality index values by station are presented in Figure 5.

Seasonal variations in water quality were evaluated using Water Quality Index (WQI) analysis for seven stations. Except for Stations 4 and 7, the stream exhibited the highest WQI values (i.e., the lowest water quality) during the autumn period, while the lowest WQI values (i.e., the highest water quality) were recorded in the spring period. For all stations other than Stations 4 and 7, the seasonal ranking of WQI values followed the order: autumn > summer > winter > spring.

Stream discharge exhibits seasonal variability. While discharge values are high during the spring, they tend to decrease in the autumn [25,114]. The high level of pollution observed in the autumn period has been associated with insufficient flow, leading to water stagnation and a consequent reduction in the self-purification capacity of the stream ecosystem [15]. Autumn is generally the period following summer drought, during which intense winter precipitation and the associated soil erosion have not yet begun. Water temperature is high during summer and early autumn. An increase in temperature enhances the solubility of pollutants and accelerates the rate of chemical reactions of pollutants in water [115]. Water at higher temperatures can hold less dissolved oxygen, and a decrease in oxygen can rapidly deteriorate the water quality [116]. During the summer, high temperatures, evaporation-induced stagnation, and low flow prevent the dilution of pollutants [84].

In spring, increased precipitation and snowmelt enhance the stream’s water volume [117]. If pollution in the basin originates from point sources (e.g., factories, treatment plants), the larger water mass dilutes it and, by altering flow velocity, can reduce pollutant concentrations [118]. Due to precipitation beginning in winter and continuing until mid-spring, the streambed is flushed with high discharge by the end of the winter period (transition to spring) [119]. At Station 4, while the stream exhibited the highest water quality during the spring period, it was observed—unlike the other stations—that the lowest quality occurred in winter. The lowest water quality in winter may be attributed to the strong influence of runoff-related pollution, whereby pollutants from agricultural areas, settlements, and industry can be transported into the water via surface flow. At Station 7, the periods of highest and lowest water quality were similar to those at the other stations, occurring in spring and autumn, respectively; however, differences were observed in the ranking of the intermediate seasonal periods. The ranking of seasonal variations in water quality, while not necessarily being the same for each station, may vary depending on the station’s location and anthropogenic activities.

### 4.2. Evaluation of One-Way ANOVA

Based on the results of the one-way ANOVA, the group means indicated that Station 1 had lower average values than the other stations. This was considered to be due to its proximity to the upstream section of the river. Pairwise comparisons among stations (Tukey) showed that Station 1 differed significantly from all other stations in water quality index values [120]. Station 7 was found to be significantly different from Stations 2 and 5. The mean water quality values of Station 7 were determined to be higher than those of Stations 2 and 5 [121]. The statistically significantly higher mean values observed at Station 7 compared to the other stations indicate that pollution pressure across the basin is concentrated at this station. This finding is supported by the station’s proximity to the downstream section of the stream [122,123].

### 4.3. Evaluation of Kruskal–Wallis Test

Based on the pairwise comparisons of the non-parametric Kruskal–Wallis test conducted using the same dataset, Station 1 was found to differ significantly from the other stations. Furthermore, its lower mean rank value compared to the other stations indicates that this station has the best water quality [124]. Although Station 7 exhibited a high mean rank value, indicating the lowest water quality, the absence of statistically significant differences between this station and the others suggests that this difference is not statistically pronounced. The absence of significant differences among the other stations suggested that they exhibited similar water quality characteristics. The Nilüfer Stream and its tributaries (Stations 2, 3, 4, 5, 6, and 7) become polluted due to industrial, urban, and agricultural pressures as they pass through the city, and they exhibit lower water quality at the point where they discharge into the sea compared to their source. When the results of the one-way ANOVA and Kruskal–Wallis tests were evaluated together, it was revealed that Station 1 is the most discriminative station in the study area, while the differences among the other stations vary depending on the method used. This situation arises from the differences in the sensitivity of parametric and non-parametric tests to data distribution.

### 4.4. Principal Component Analysis 

In the principal component analysis conducted using meteorological parameter data, the variables were grouped into two main components. PC1 was interpreted as representing general atmospheric conditions and phase changes in water, whereas PC2 was interpreted as representing atmospheric dynamics and solid precipitation processes. Relative humidity affects air temperature and pressure, while air temperature, pressure, and wind speed influence evaporation [125,126]. Changes in the frequency, intensity, duration, and spatial distribution of precipitation associated with climate change affected water regimes, surface runoff, erosion processes, pollutant concentrations, and dissolved oxygen levels, thereby leading to variations in water quality [118,127]. Hydrological processes shape with the basin’s physical structure and with human interventions interacted with changes in the balance between precipitation and evaporation, thereby affecting streamflow Dynamics [128]. Furthermore, meteorological parameters (air temperature, relative humidity, precipitation, wind speed, etc.) significantly affected water quality parameters [4,9,129]. Changes in atmospheric pressure, driven by climate change, alter wind patterns and pressure systems. These changes, in turn, contribute to extreme weather events such as droughts, floods, and inundations [130]. On the other hand, seasonal increases in temperature, together with high conductivity conditions, reduce the adsorption capacity of metals, affecting their dissolution processes and leading to their release into the water body [131].

In the principal component analysis conducted using meteorological parameters and water quality index data from the stations, five main components were obtained (Table 6). The analysis demonstrated that water quality in the Nilüfer Stream and meteorological parameters were not governed by a single dominant control mechanism, but rather exhibited a multidimensional structure shaped by both spatial and anthropogenic influences. Although WQI-St 1, WQI-St 2, and WQI-St 6 showed similar loadings in PC1, their WQI values differed. This situation suggests that the stations responded in a similar way to the environmental variations represented by PC1; however, it is thought that their absolute water quality conditions were separated due to differences in anthropogenic pressure levels. PC2, with positive loadings for actual pressure and snow depth and negative loadings for temperature, represented winter-season atmospheric conditions and indicates a seasonal structure. Atmospheric pressure variations indirectly affect snow depth by controlling precipitation formation and type, while changes in air temperature influence snow cover depth through melting processes [132,133,134,135]. PC3, with positive loadings for precipitation and relative humidity and a negative loading for evaporation, represented a hydro-meteorological process. Precipitation, relative humidity, and WQI-St 4 were positively correlated, whereas evaporation showed an inverse relationship [136]. The inclusion of WQI-St 4 in this component has been thought to indicate that this station is sensitive to natural flow and precipitation regimes. At Station 4, precipitation and relative humidity are changing nutrient transport via surface runoff, while evaporation is changing the dilution capacity of the stream [10]. Therefore, pollutant concentration levels are affected. At Station 4, there is no large Organized Industrial Zone. For this reason, small-flow wastewater discharges enter the stream. This situation may have caused the effect of climatic parameters, together with wastewater inputs, to be observed. PC4, with a positive loading for wind speed and a negative loading for WQI-St 7, indicates that this station exhibits a different response to atmospheric transport processes and is inversely affected by wind-related local atmospheric influences. Wind is one of the most important meteorological parameters affecting hydrodynamic processes. It also causes an increase in the distribution of turbidity and pollutants within the stream [9]. Station 7 is the last station in the study area, i.e., it is located in the downstream section. Therefore, it is in an area with fewer settlements. This situation leads to a stronger influence of wind being felt. The low building density causes air flows to encounter fewer obstacles and causes higher wind speeds to be observed [137]. PC5, with WQI-St 3 and WQI-St 5 loading in the same direction, has been thought to suggest that these stations may exhibit similar water quality dynamics under similar anthropogenic impact conditions, despite being located in different basins. The fact that both stations are located downstream of wastewater treatment plant discharge points (EWWT and WWWT) indicates that this similarity may be associated with point-source pollution inputs. Overall, the PCA results indicated that water quality variables are not strongly and directly integrated with meteorological parameters; instead, the stream water quality was structured by both spatial location and anthropogenic pressures. Meteorological variables are predominantly understood to represent seasonal atmospheric processes and are considered to play an indirect and regulatory role rather than a direct controlling influence on water quality.

The compounds forming the first principal component are the parameters that vary most together, i.e., those showing a strong degree of correlation [106]. Since interrelated (correlated) variables receive higher loadings within the same principal component, these variables can be interpreted as a group and may help to identify environmental processes or factors [138]. PCA results from seven stations using water quality parameters were evaluated. As Station 1 was located near the upstream section of the Nilüfer Stream and is generally surrounded by urban residential areas, domestic wastewater reaches this station. Therefore, the fact that the most strongly associated parameters in PC1 were heavy metals was attributed not directly to industrial activities, but rather to the contribution of metals from natural formations and urban influences (e.g., traffic load, domestic wastewater, detergents, etc.) entering the stream. Various factors such as weather conditions, rainfall frequency, and traffic intensity (vehicle exhaust emissions, tire and brake wear) affect heavy metal concentrations in urban basins, and runoff from impervious surfaces directly reaches the stream [139]. In addition, atmospheric deposition, agricultural activities, and geological background may also act as pollution sources [140,141]. PC2 showed a negative loading for pH, which can be explained by increased organic matter associated with urban activities and, consequently, enhanced oxygen consumption through microbial processes, leading to increased water acidity [142,143]. PC3 was explained by positive loadings of TN and TP and a negative loading of dissolved oxygen, with increased oxygen consumption resulting from higher organic matter content associated with elevated phosphate from urban wastewater and nitrate from agricultural activities [144]. PC4 included EC, which may serve as an indicator of mineral pollution due to its relationship with ionized substance concentration [75]. This component may also represent natural pollution arising from processes such as seasonal variations and rock weathering [145]. Overall, the results of the analysis were associated with both natural formations and pollution derived from urban and agricultural activities. In a study conducted by Yolcu (2012) [122] on the Nilüfer Stream, a decreasing trend in water quality was identified for BOD and COD parameters, and an increase was predicted not only for conventional parameters but also for heavy metals. Station 1 is located in Gümüştepe. There are no industrial facilities or manufacturing plants within Gümüştepe. In addition, agricultural land is located in the vicinity of Gümüştepe.

At Station 2, three main components were identified, and PC1 was interpreted as representing domestic, industrial and agricultural pollution. Zinc (Zn) is transported to aquatic systems via surface runoff associated with rainfall events originating from industrial activities as well as domestic waste and agricultural practices (fertilizers and pesticides), and through atmospheric deposition from traffic-related emissions, along with the leaching of particles derived from tire and road wear [146,147,148,149]. This finding indicated mixed anthropogenic sources, including urban and industrial discharges as well as agricultural activities (fertilizers, etc.). PC2 was interpreted as representing the metal load of the water. The dominance of heavy metals suggested a common pollution source and indicated industrial discharges as a potential contributor [150]. Furthermore, it is known that the effluent discharge from the wastewater treatment plant operated by the S.S. Yeşil Çevre Services and Management Cooperative, which collects domestic and industrial wastewater from the enterprises operating in the Gürsu (Uludağ) OIZ, Kestel OIZ, Barakfakih OIZ, and Kestel Industrial Area, as well as from the residential areas of Kestel and Gürsu, reaches this station. PC3 was interpreted as reflecting physicochemical control mechanisms rather than direct pollution sources. The solubility of elements in aquatic environments is governed by pH, while the oxidation–reduction potential controls oxygen balance and biochemical reaction processes in the system [118]. The 3rd station is located on the Deliçay stream and is downstream of the discharge from the East WWTP. In addition to the sources identified at Station 2, the discharge from the East Wastewater Treatment Plant, which treats all domestic wastewater originating from the eastern part of the city as well as domestic and industrial wastewater from the Demirtaş Organized Industrial Zone (OIZ), also reaches this station. However, the amount of water quality data available for this station was lower than that of the other stations. PC1 represented the heavy metal load of the water. The presence of Pb, Cd, Cu, and Ni within the same component suggests that these metals likely originate from a common source, namely industrial activities [151,152]. Furthermore, treated wastewater discharges from the Demirtaş OIZ wastewater treatment plant also reach this station. The grouping of metals at this station was found to be consistent with the metal group observed at the 2nd station (Cd, Ni, Cu, Pb), which is located on the same tributary. PC2 reflected physicochemical characteristics and organic pollution. A decrease in dissolved oxygen was observed due to the decomposition of organic matter and the increase in dissolved ions [153]. At the same time, variations in water temperature increase ion mobility while limiting gas solubility, leading to reduced dissolved oxygen levels [154]. Due to the Bursa East Wastewater Treatment Plant, the organic pollution load discharged into this area is lower compared to Station 2. Therefore, in terms of WQI, it has been found to have a lower value than Station 2 and thus to be less polluted. PC3 reflected pollution associated with domestic, industrial wastewater and agricultural drainage sources. In this component, Zn was dominant with a high loading, while other parameters showed moderate loadings; this situation suggests that they do not exhibit strong co-variation at high levels, but their presence in water is influenced by similar factors [10]. Domestic wastewater and agricultural practices (e.g., fertilizers) contribute to Zn concentrations in water [155]. Stations 2 and 3 are located on the Deliçay Stream, where the surrounding area is dominated by various industries, predominantly textile manufacturing, as well as dyeing, chemical, metal processing, and food processing. These industrial sectors are known to be associated with metals such as Ni, Pb, Zn, Cd, and Cu.

Station 4 is located on the Ayvalı Stream, upstream of the discharge from the West Wastewater Treatment Plant. Domestic and industrial wastewater originating from small and medium scale industrial facilities in the Çalı, Yaylacık, and Ürünlü areas reaches this station. PC1 represented metal pollutants showing similar variation patterns. These metals may originate from urban influences or industrial activities (e.g., iron and steel production, metal processing, paint, textile, etc.) [156,157]. PC2 reflected the organic matter and nutrient load of the water. It indicated the influence of domestic wastewater discharges and surface runoff associated with agricultural activities. The influence of agricultural practices was observed at this station. PC3 could be explained as the relationship between ionic and metallic load and dissolved oxygen. The separate clustering of Zn from other metals was thought to suggest that this metal is introduced into the stream through different anthropogenic pathways. The negative loading of dissolved oxygen indicates that metals become more soluble or mobile under low-oxygen conditions [10]. PC4 represented variations in pH and Ni resulting from domestic and industrial wastewater inputs. The Ni parameter represented metal accumulation originating from region-specific industrial sources. The mobility of metals in water environments is largely controlled by ambient pH [158]. At Station 5, located on the Ayvalı Stream after the discharge from the West wastewater treatment plant, PC1 was interpreted as representing the organic, nutrient, and metal components of the water. It indicated domestic and industrial discharges as well as agricultural sources. In addition, this area, located near the Hamitler solid waste landfill site, contains large amounts of nitrogenous organic matter, and leachate waters may enter streams after being treated in wastewater treatment plants [107]. PC2 was interpreted as representing the heavy metal load of the water. The strong correlations among these metals suggested that they may originate from similar sources, such as industrial activities. PC3 was interpreted as a physicochemical and metal pollution component affecting water quality. The strong loading of electrical conductivity reflected the ionic conditions of the water or pollution from urban and industrial sources, and, as previously noted, dissolved oxygen influenced the solubility and mobility of metals. The moderate loading of Ni might suggest the influence of localized industrial activities. The weak loading of pH indicates that the explanatory power of this parameter for the component is limited [159]. This area is located downstream of the Bursa West Wastewater Treatment Plant, where all domestic wastewater from the western part of the city, as well as domestic and industrial wastewater from Nilüfer OIZ and Bursa OIZ, are discharged into the region. Therefore, both organic and metal pollution are observed. These organized industrial zones include automotive, metallurgy, metal-processing, and textile industries. These industries are often associated with high levels of metals such as Ni, Zn, Pb, Cd, and Cu. In addition, it would be appropriate to consider the effects of diffuse pollution from agricultural activities as well as the potential impacts of the metal processing industry.

At Station 6 on the Hasanağa Stream, PC1 was interpreted as representing the organic matter and nutrient load of the water. It indicated domestic and industrial wastewater discharges. An inverse relationship was observed between dissolved oxygen and pH, and the organic matter and nutrient load. PC2 could be interpreted as reflecting the water’s heavy metal load. As in other stations, these metals (Cu, Cd, Ni, Pb) generally showed similar trends, suggesting that they may originate from similar industrial activities. PC3 could be interpreted as representing the nutrient and metal load of the water. These parameters might be influenced by a common source (e.g., agricultural activities) or by similar processes. This station is located at a location where industrial and domestic wastewater discharges from Kayapa OIZ, Hasanağa OIZ, and various industrial facilities reach the area [122]. Wastewater from the Kayapa OIZ is treated at the Hasanağa OIZ wastewater treatment plant and discharged into the Hasanağa stream. It also suggested impacts from industrial sectors (metal, machinery, food, etc.) and agricultural sources (fertilizers, pesticides, etc.). For Station 7, PC1 could be interpreted as representing the metal and nutrient load of the water. Metal loads generally indicate influences from industrial and urban sources, while nutrient loads reflect impacts from wastewater and surface runoff. PC2 could be interpreted as representing the organic matter and nutrient load of the water. It suggested oxygen related variations driven by organic matter and nutrient inputs from industrial and domestic discharges. PC3 represented the metal load of the water. These metals predominantly suggested the influence of industrial and urban sources. PC4 could be interpreted as reflecting the acid–base condition of the water [80]. Therefore, as previously mentioned, it plays an important role in water quality. All wastewater discharges from the city reach this station. Therefore, high levels of pollution were observed at this station. As the final monitoring station before the discharge of the Nilüfer Stream into the sea, this site is important for representing the overall pollution load of the stream. Station 6 is located on Hasanağa Stream, while Station 7 is located downstream on the main channel of the stream after the confluence of Hasanağa Stream and Nilüfer Stream. The Hasanağa Stream watershed is dominated by automotive, machinery, metal-processing, iron and steel, paper, chemical, and plastics industries. These sectors are commonly associated with heavy metals, particularly Zn, Pb, Cu and Ni. The r values obtained from Spearman correlation analysis were found to be generally consistent with the PCA analyses for all stations. Similar consistency was also observed in different studies [160].

In the Nilüfer Stream, it has been observed that pollutants originating from industrial activities and from surface runoff in urban areas adversely affect water quality. According to the PCA results, sites with high pollution levels were mostly those located within urban areas or directly influenced by industrial wastewater discharges. Turkey is geographically located in the mid-latitudes and therefore experiences four distinct seasons. Diverse weather conditions, factors such as precipitation amount and frequency, and traffic density and similar factors, together influence heavy metal concentrations in urban basins [139].

The comparison of the present study with similar studies was provided in Table 7. Due to differences in parameter sets and weighting schemes, caution should be taken when making numerical comparisons between the water quality index (WQI) obtained in this study and other global indices. In a study conducted on the Swat River in northern Pakistan (2020) [75], water quality was assessed using the WQI, and a similar classification scheme to the present study was applied. The study reported that WQI values ranged from 13.58 to 209, indicating low water quality for drinking and domestic purposes. Pollution levels were associated with both natural and anthropogenic sources (agricultural and industrial activities). Dibal and Olomukoro (2025) [161] evaluated the water quality of the Gongolo River and obtained water quality results close to those of the present study. It was determined that the water quality index (WQI) values ranged from very poor (93.38) to unsuitable (109.21), and that water pollution could originate from both natural sources and anthropogenic activities. In a study conducted by Li et al. [162], WQI values of the Haihe River ranged from 60 to 90, indicating that the river had moderate to good water quality. It was reported that the dominant pollutant originating from anthropogenic activities such as agricultural runoff and urban wastewater was organic matter, with similar contributions from nutrients. It was observed that the water quality of the river was better compared to the present study. Similarly, in a study conducted by Luo et al. [163] in the Qiantang River Basin, the average water quality index (WQI) was 79.26, and the water quality was evaluated as good. It was emphasized that pollution sources were closely related to regional land use patterns, and that built-up areas and intensifying agricultural lands were the main sources of pollution.

## 5. Conclusions

The following results were obtained in this study:

Water quality was converted into a single numerical value using WQI, thus allowing the combined evaluation of many water quality parameters. In terms of regional variation, the upstream and downstream sections differed from the other stations, whereas the other stations did not differ from one another due to high pollution loads. In addition, it was observed that the upstream section was suitable for irrigation, while the other stations could only be suitable for industrial usage under controlled conditions.

While the PCA conducted among water quality parameters showed that the relationships were stronger among parameters mainly originating from industrial wastewater, the groups formed at Station 1 indicated the presence of urban and agricultural pollution.

PCA of meteorological parameters and stations’ WQI values showed that climate change may be effective only at two stations. It was determined that precipitation, humidity, and evaporation were related in the area located before the wastewater treatment plant, while the downstream section was associated with wind speed. It was considered that precipitation may affect concentrations by altering flow, and that wind speed may affect concentrations by altering flow velocity and dispersion. In addition, it was determined that air temperature, evaporation, relative humidity, actual pressure, and precipitation amount formed a more closely related group, while snow depth and wind speed also formed another such group. It was understood that the stream is more affected by industrial, urban, and agricultural pollution sources than by climate change.

It was observed that the use of multivariate statistical methods together with climate change parameters provides a more accurate and holistic evaluation for determining the water quality of streams carrying high pollution loads. Thus, pollution sources can be evaluated more effectively, enabling the development of future sustainable integrated watershed management strategies.

## Figures and Tables

**Table 1:** Sampling stations and features.

Station No	Features of the Study Area	Coordinates
1	On the Nilüfer Stream, Gümüştepe area (Main stem and headwater region)	40°10′41″ N 28°58′11″ E
2	On the Deliçay Creek, before the discharge from the East Wastewater Treatment Plant (On the Deliçay Creek, a tributary of the Nilüfer Stream)	40°14′17″ N 29°06′42″ E
3	On the Deliçay Creek, after discharge from the East Wastewater Treatment Plant (On the Deliçay Creek, a tributary of the Nilüfer Stream)	40°14′21″ N 29°04′27″ E
4	On the Ayvalı Creek, before the discharge from the West Wastewater Treatment Plant (On the Ayvalı Creek, a tributary of the Nilüfer Stream)	40°12′30″ N 28°53′05″ E
5	On the Ayvalı Creek, after the discharge from the West Wastewater Treatment Plant (On the Ayvalı Creek, a tributary of the Nilüfer Stream)	40°14′42″ N 28°54′36″ E
6	On the Hasanağa Creek (On the Hasanağa Creek, a tributary of the Nilüfer Stream)	40°11′10″ N 28°48′03″ E
7	On the Nilüfer Stream, after the confluence with the Hasanağa Creek (Main stem and downstream region)	40°15′40″ N 28°47′43″ E

**Table 2:** Mean and standard deviation values of the meteorological parameters of Bursa Province for the period 2008–2024.

Parameters	Mean ± Standard Deviation
Monthly average of total precipitation (mm)	1.9018 ± 2.3607
Monthly average wind speed (m/s)	2.2212 ± 0.4241
Monthly average relative humidity (%)	70.4648 ± 7.7450
Monthly average of total snow depth (cm)	0.7223 ± 2.2255
Monthly average pressure (hPa)	1003.6794 ± 3.8248
Monthly average of total evaporation (mm)	4.0378 ± 2.0393
Monthly average air temperature (°C)	15.7165 ± 7.0910

**Table 3:** Mean and standard deviation values of the water quality parameters for the period 2008–2024.

Parameters	Mean ± Standard Deviation											
	Station 1	Station 2	Station 3	Station 4	Station 5	Station 6	Station 7	EPA *	WHO *	Turkish Standard (TSWQR)		
	(726 Data)	(726 Data)	(440 Data)	(737 Data)	(616 Data)	(693 Data)	(748 Data)			Class I	Class II	Class III
pH	8.32 ± 0.46	8.03 ± 0.46	7.82 ± 0.40	7.89 ± 0.68	7.68 ± 0.46	7.56 ± 0.45	7.67 ± 0.41	6.50–9.00	6.50–8.00	6.00–9.00	6.00–9.00	6.00–9.00
EC (microS/cm)	460.2 ± 161.7	1720.8 ± 1038.6	1576.3 ± 501.4	1621.9 ± 1146.7	1551.7 ± 588.3	1728.4 ± 806.8	1428.0 ± 535.3	-	-	<400	1000	>1000
DO (mg/L)	9.94 ± 1.90	4.56 ± 3.25	4.31 ± 2.53	5.73 ± 3.74	5.92 ± 2.33	3.72 ± 2.57	2.67 ± 1.99	-	-	>8	6	<6
TN (mg/L)	1.550 ± 1.205	10.401 ± 7.283	9.479 ± 5.674	17.474 ± 13.800	12.723 ± 10.918	10.426 ± 7.630	14.998 ± 7.655	-	-	<3.5	11.5	>11.5
TP (mg/L)	0.089 ± 0.062	1.463 ± 0.977	1.741 ± 1.023	1.189 ± 1.140	1.432 ± 1.392	1.576 ± 0.904	2.406 ± 1.581	-	-	<0.08	0.2	>0.2
COD (mg/L)	13.77 ± 7.26	193.37 ± 152.26	145.17 ± 83.66	111.99 ± 151.17	94.38 ± 118.19	187.63 ± 166.10	133.22 ± 79.07	-	-	<25	50	>50
Pb (mg/L)	0.0400 ± 0.0457	0.0360 ± 0.0442	0.0242 ± 0.0233	0.0417 ± 0.0519	0.0467 ± 0.0621	0.0347 ± 0.0436	0.0393 ± 0.0444	0.0025	0.01	0.014		
Ni (mg/L)	0.0303 ± 0.0341	0.0331 ± 0.0342	0.0325 ± 0.0276	0.1110 ± 0.1602	0.0798 ± 0.0508	0.0338 ± 0.0264	0.0621 ± 0.0458	0.052	0.07	0.034		
Zn (mg/L)	0.0659 ± 0.0935	0.2982 ± 0.3440	0.2198 ± 0.1691	0.5397 ± 0.9837	0.4851 ± 0.7381	1.1956 ± 6.0672	0.3402 ± 0.3689	0.12	-	0.231		
Cu (mg/L)	0.0263 ± 0.0373	0.0328 ± 0.0345	0.0249 ± 0.0276	0.0356 ± 0.0402	0.0304 ± 0.0365	0.0247 ± 0.0269	0.0408 ± 0.0427	-	2	0.0031		
Cd (mg/L)	0.0184 ± 0.0342	0.0190 ± 0.0351	0.0152 ± 0.0247	0.0181 ± 0.0340	0.0194 ± 0.0370	0.0135 ± 0.0207	0.0180 ± 0.0337	-	0.003	0.0015		

* EPA: Environmental Protection Agency Aquatic Life Criteria. * WHO: World Health Organization Drinking Water Criteria.

**Table 4:** Seasonal and overall water quality index (WQI) values for each station.

Station	Spring	Summer	Autumn	Winter	Overall
1	121.49	135.80	173.01	132.62	140.83
2	214.47	284.18	502.47	274.29	321.96
3	265.03	337.47	364.33	310.67	317.36
4	221.09	333.49	421.75	436.61	348.23
5	232.99	346.19	446.40	336.03	345.40
6	232.01	330.55	426.64	325.49	329.96
7	315.11	396.77	569.86	466.32	437.83

**Table 5:** Results of the one-way ANOVA analysis.

ANOVA					
WQI	Sum of Squares	Df	Mean Square	F	Sig.
Between Groups	12.008	6	2.001	34.900	0.000
Within Groups	24.028	419	0.057		
Total	36.036	425			

**Table 6:** Results of the principal component analyses applied to meteorological parameters, water quality parameters, and the combined meteorological parameters and water quality index (WQI) values and spearman correlation analysis.

	Component	Total	Parameters		% Variance	Cumulative %
Meteorological Parameters	1	3.546	Air temperature, Evaporation, Relative		50.599	50.599
			humidity, Actual pressure, Precipitation			
	2	1.274	Snow depth, Wind speed		18.261	68.86
Meteorological Parameters and WQI values	1	3.742	WQI-St* 1, WQI-St* 2, WQI-St* 6		21.345	21.345
	2	3.005	Actual pressure, Snow depth, Air		20.818	42.163
			temperature			
	3	1.899	Precipitation, Relative humidity,		17.654	59.818
			Evaporation, WQI-St* 4			
	4	1.216	WQI-St* 7, Wind speed		9.729	69.546
	5	1.028	WQI-St* 3, WQI-St* 5		8.239	77.785
**Station No**	**Component**	**Total**	**Parameters**	**Spearman Correlation—r Value**	**% Variance**	**Cumulative %**
1	1	4.437	Cu, Ni, Cd, Pb, Zn	0.796–0.521	34.953	34.953
	2	2.000	pH, COD		16.728	51.681
	3	1.134	DO, TN, TP	0.350–0.320	15.804	67.485
	4	1.043	EC		10.822	78.307
2	1	3.672	TP, COD, Zn, EC, DO, TN	0.720–0.269	31.668	31.668
	2	3.258	Cd, Ni, Cu, Pb	0.625–0.322	30.697	62.365
	3	1.169	pH		11.253	73.618
3	1	3.856	Pb, Cd, Cu, Ni	0.697–0.347	30.933	30.933
	2	3.224	DO, EC, COD, pH	0.723–0.334	26.669	57.602
	3	1.019	Zn, TP, TN	0.443–0.338	16.022	73.624
4	1	3.383	Cu, Pb, Cd	0.574–0.403	22.284	22.284
	2	2.477	TP, COD, TN	0.614–0.326	20.869	43.153
	3	1.201	DO, EC, Zn	0.673–0.493	18.763	61.917
	4	1.11	pH, Ni	0.249	12.361	74.278
5	1	4.068	COD, TN, Zn, TP	0.602–0.269	26.613	26.613
	2	2.299	Cu, Cd, Pb	0.621–0.458	25.148	51.76
	3	1.173	EC, DO, Ni, pH	0.607–0.305	16.777	68.538
6	1	3.202	COD, TP, DO, pH, EC	0.702–0.346	27.848	27.848
	2	2.666	Cu, Cd, Ni, Pb	0.564–0.312	23.736	51.584
	3	1.298	Zn, TN	0.523	13.551	65.135
7	1	3.632	Zn, Ni, TP	0.568–0.375	23.294	23.294
	2	2.549	EC, COD, DO, TN	0.622–0.381	22.754	46.048
	3	1.331	Cd, Pb, Cu	0.568–0.304	21.074	67.122
	4	1.155	pH		11.667	78.789

* WQI-St: The WQI value of the station.

**Table 7:** Comparison of the Present Study with Similar Studies.

Study Area	Parameters	WQI Rank	PCA/Dominant Components	Referance
Swat River	pH, electrical conductivity (EC), and total dissolved solids (TDS), chloride (Cl), sulfate (SO4), bicarbonate alkalinity (HCO3), Na, K, Ca, Mg, Zn, Co, Cu, Ni, Pb	13.58–209	Both naturally and anthropogenically	[75]
Gongola River	pH, EC, TDS, Nitrate, Phosphate, Turbidity, TH, Alkalinity, BOD, DO, TSS	93.38–109.21	Anthropogenic (especially agricultural activities) and natural sources (EC, TDS, TH, Turbidity, TSS, BOD)	[161]
Haihe River	pH, ammonia nitrogen (NH_3_-N), Biochemical oxygen demand (BOD), Chemical oxygen demand (COD), Dissolved oxygen (DO), total phosphorus (TP)	60–90	Organic matter and nutrient salts (COD, BOD5, NH3-N ve TP)	[162]
Qiantang River Basin	pH, dissolved oxygen (DO), permanganate index (CODmn), five-day biochemical oxygen demand (BOD5), ammonia nitrogen (NH3-N), total nitrogen (TN), total phosphorus (TP)	79.26	Nitrogen and phosphorus pollution and organic pollutants (TN, TP, NH3-N, COD, BOD5.)	[163]
Nilüfer Stream	pH, İletkenlik, Çözünmüş oksijen, KOİ, TN, TP, Pb, Ni, Cu, Cd, Zn	140.83–487.83	Kentsel deşarjlar, endüstriyel deşarjlar, tarımsal faaliyetler	This Study

## Data Availability

The raw data supporting the conclusions of this article will be made available by the authors on request.

## Supplementary Materials

The following supporting information can be downloaded at: https://www.mdpi.com/article/10.3390/toxics14060520/s1, Figure S1: Scree plot of principal component analysis for meteorological parameters and stations’ WQI values; Figure S2: Scree plots of principal component analysis for water quality parameters at the stations (Station 1 (a), Station 2 (b), Station 3 (c), Station 4 (d), Station 5 (e), Station 6 (f), Station 7 (g)).

## Abbreviations

The following abbreviations are used in this manuscript:

St	Sampling Station
WQI	Water Quality Index
PCA	Principal Component Analysis
OIZ	Organized Industrial Zone
WWWTP	West Wastewater Treatment Plant
EWWTP	East Wastewater Treatment Plant
ANOVA	Analysis of Variance
TSWQR	Turkish Surface Water Quality Regulation
