# Supplementary figures and images for: Assessing the Water Quality of a Stream and Its Relationship with Climate Change Using Water Quality Index and Multivariate Statistical Methods
Source: Toxics. 2026 Jun 15;14(6):520. doi: 10.3390/toxics14060520 (PMC13307280; doi:10.3390/toxics14060520)

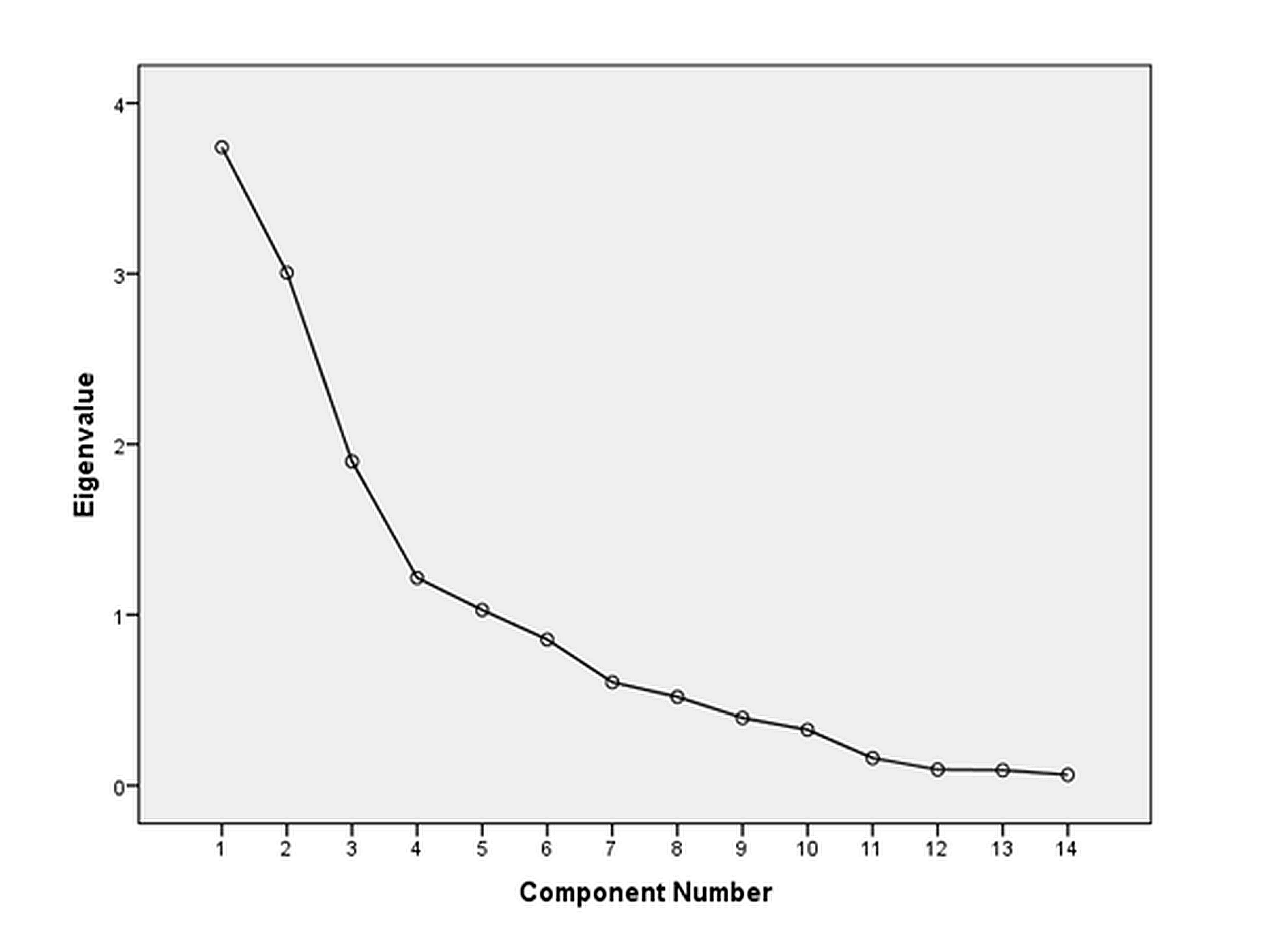

Supplement: Supplementary file 1 [file toxics-14-00520-s001.zip › Figure S1.tif]

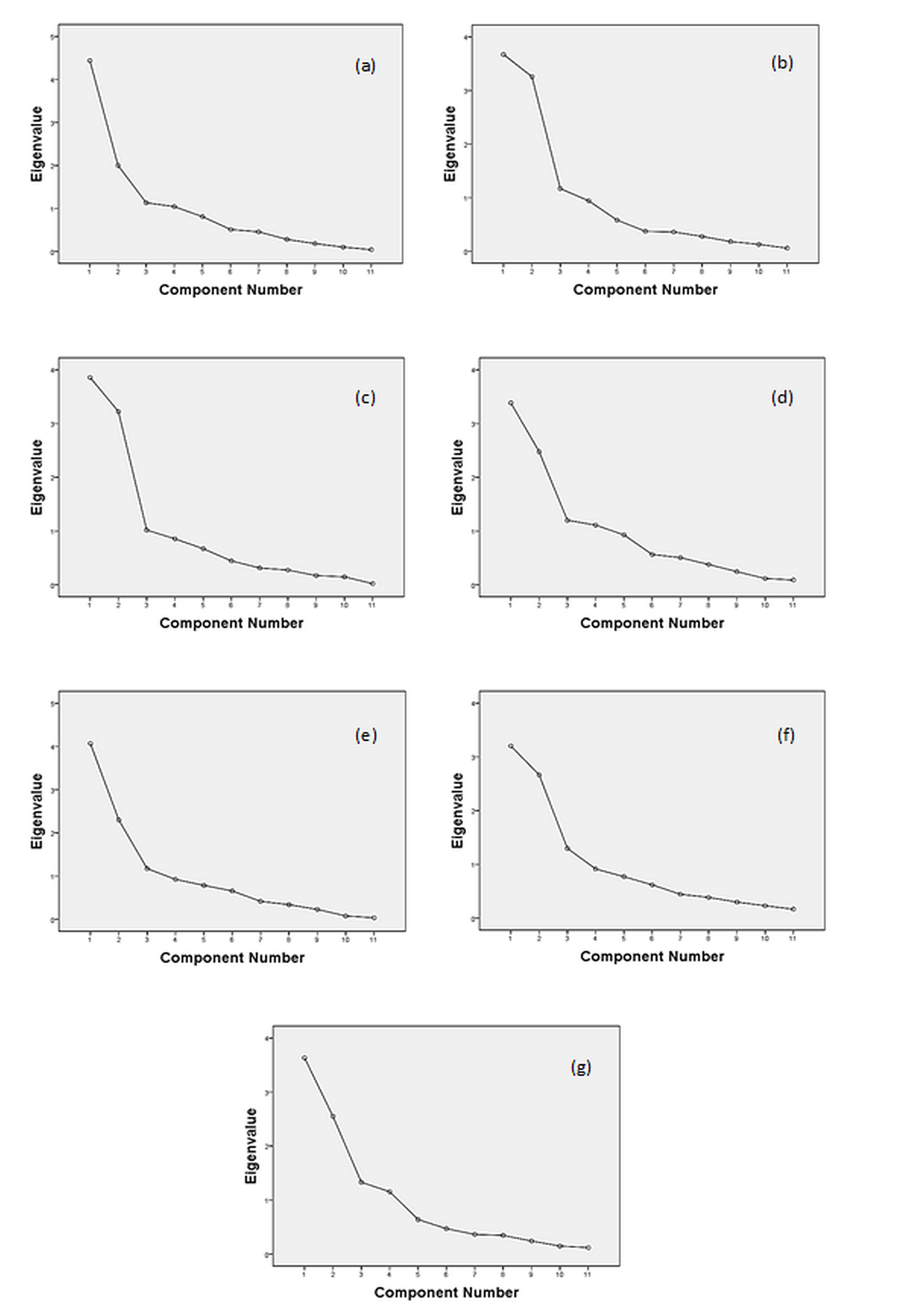

Supplement: Supplementary file 1 [file toxics-14-00520-s001.zip › Figure S2.tif]
